# Heart in a dish – choosing the right *in vitro* model

**DOI:** 10.1242/dmm.049961

**Published:** 2023-02-24

**Authors:** Lika Drakhlis, Robert Zweigerdt

**Affiliations:** Leibniz Research Laboratories for Biotechnology and Artificial Organs (LEBAO), Department of Cardiothoracic, Transplantation and Vascular Surgery (HTTG), REBIRTH - Research Center for Translational Regenerative Medicine, Hannover Medical School, Hannover 30625, Germany

**Keywords:** Heart, Development, Therapeutic, Pluripotent stem cells, Organoids, Gastruloids, Cardiac tissue engineering, Congenital disease modeling, Microtissues, Drug screening

## Abstract

The heart is the first functional organ established during embryogenesis. Investigating heart development and disease is a fascinating and crucial field of research because cardiovascular diseases remain the leading cause of morbidity and mortality worldwide. Therefore, there is great interest in establishing *in vitro* models for recapitulating both physiological and pathological aspects of human heart development, tissue function and malfunction. Derived from pluripotent stem cells, a large variety of three-dimensional cardiac *in vitro* models have been introduced in recent years. In this At a Glance article, we discuss the available methods to generate such models, grouped according to the following classification: cardiac organoids, cardiac microtissues and engineered cardiac tissues. For these models, we provide a systematic overview of their applications for disease modeling and therapeutic development, as well as their advantages and limitations to assist scientists in choosing the most suitable model for their research purpose.

## Teaser

**Summary:** We provide a systematic overview of currently available *in vitro* cardiac models, their method of generation, properties and examples for applications. Moreover, each model's advantages and limitations are discussed.

## Introduction

Cardiovascular diseases are the leading cause of death worldwide (Virani et al., 2020). Although extensive resources are being invested in the study of such disorders and in the development of respective treatment options, effective therapies to reverse heart failure are scarce. One of the underlying reasons is the low predictive value of the commonly used models used in preclinical research (Eder et al., 2016). Although animal models such as mouse, chicken and zebrafish have proven essential for studying the basic principles of cardiogenesis (Brade et al., 2013; Buckingham et al., 2005; Kirby, 2007; Bornhorst and Abdelilah-Seyfried, 2021), their capability to recapitulate human-specific aspects of heart development, histogenesis and physiology is limited. Apart from ethical considerations, high cost and time demands associated with animal models, a major drawback is the interspecies variability at the physiological and genetic levels, which limits the translatability of respective findings to the treatment of humans (Brandão et al., 2017; Davis et al., 2011a,b; Wobus and Löser, 2011). Therefore, there is great interest in establishing *in vitro* models that sufficiently recapitulate human heart tissue composition, structure, development and function. To comply with this need, a broad range of cardiac *in vitro* models derived from human pluripotent stem cells (hPSCs), including human embryonic stem cells (hESCs) and human induced PSCs (hiPSCs), has been established over the past years, applying different experimental approaches. In this At a Glance article and the accompanying poster, we provide a systematic overview of recent *in vitro* models of heart development and cardiac tissue, focusing on hPSCs but also including mouse pluripotent stem cell (PSC) approaches where appropriate. We give examples on how such models, which have achieved a substantial level of multi-tissue complexity, are generated and applied for investigating cardiac tissue function, disease modeling and therapeutic development, while highlighting their respective advantages and limitations.

**Figure:**
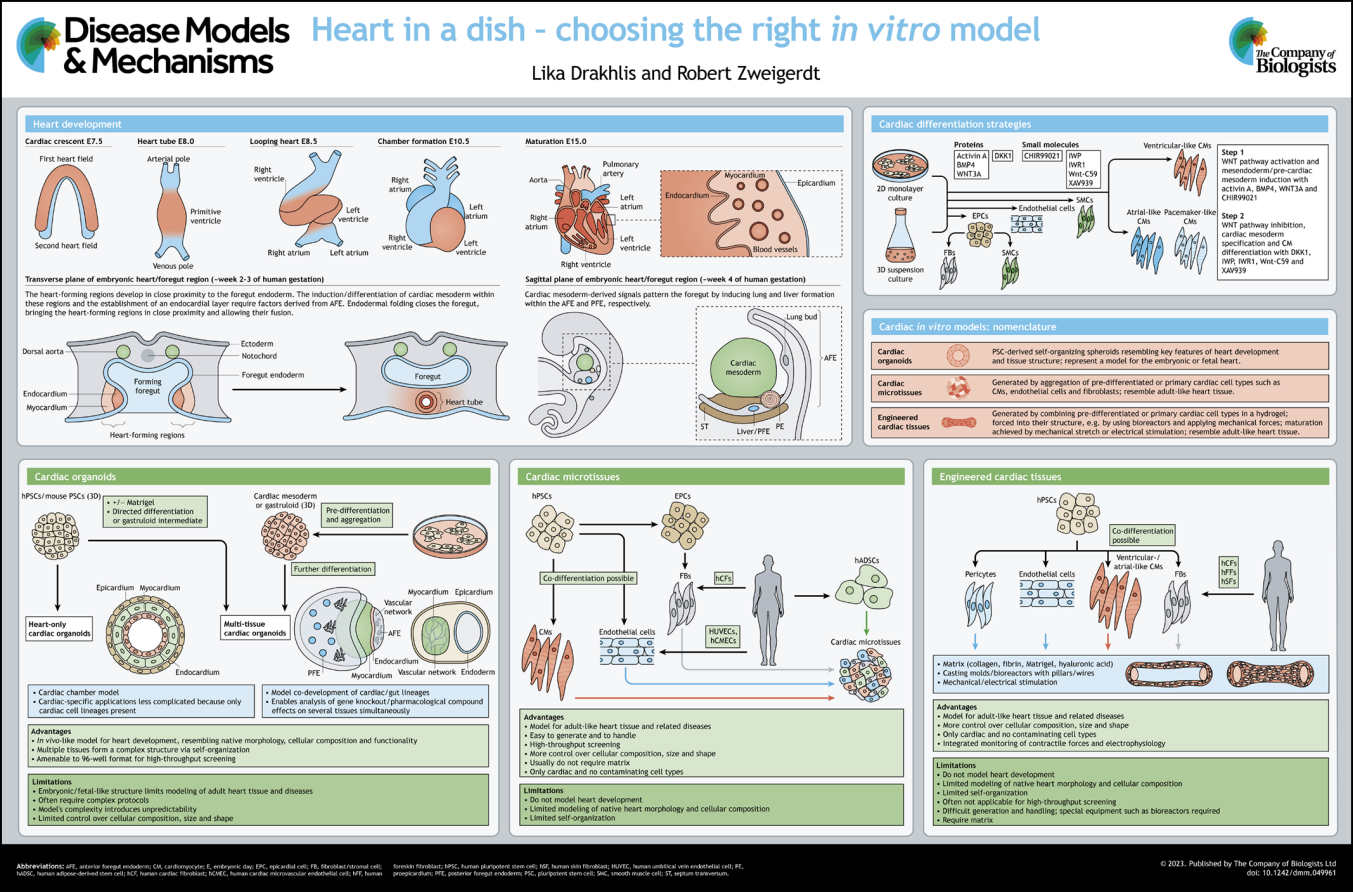


## Cardiac development

As PSC-derived models recapitulate developmental processes, refinement of these models relies on a thorough understanding of cardiac development. The heart is the first functional organ formed in the embryo (Buckingham et al., 2005). It originates from the splanchnic mesoderm, which emerges from the primitive streak during gastrulation (Garcia-Martinez and Schoenwolf, 1993). After pre-cardiac mesoderm specification in the primitive streak, these progenitors migrate in an anterolateral direction to form two groups of cells, termed the heart-forming regions, within the splanchnic mesoderm on each side of the embryonic midline. This occurs at embryonic day (E)6.5 in the mouse, corresponding to approximately week 2 of human gestation (Brade et al., 2013; Brand, 2003). Two cardiac progenitor populations, which arise from the heart-forming regions, contribute to heart formation – the first heart field (FHF) and the second heart field (SHF). The FHF, which contributes to the future left ventricle and the atria, extends across the midline to form the so-called cardiac crescent at E7.5 in the mouse or week 2–3 of human gestation. The cardiac crescent fuses at the midline into the beating, linear heart tube, which consists of two layers – the myocardium and the inner lining of the heart, the endocardium – by murine E8.0 or week 3 of human gestation (Brade et al., 2013; Srivastava, 2006; Xin et al., 2013). Whereas cells of the FHF already differentiate at the cardiac crescent stage, SHF cells remain in a multipotent, proliferative progenitor state until they are added to the heart tube at each pole, leading to its elongation (Brade et al., 2013; Srivastava, 2006). At the arterial pole, the SHF cells eventually give rise to the right ventricle and the outflow tract, and at the venous pole, the SHF contributes to the atria (Dyer and Kirby, 2009; Xin et al., 2013). In a complex process, the heart tube performs rightward looping that positions the inflow and outflow segments, as well as the future cardiac chambers (Kidokoro et al., 2022; Manner, 2013). Further remodeling and heart maturation involve septation of the ventricles and atria, as well as heart valve formation by E15.0 corresponding to roughly week 7 in humans (see poster, ‘Heart development’) (Xin et al., 2013; Brade et al., 2013).

Apart from the FHF and SHF, two other progenitor populations contribute to the heart: the proepicardium and cardiac neural crest cells. The latter give rise to smooth muscle cells of the distal outflow tract and form the aorticopulmonary septum, as well as the intracardiac nervous system of the heart. The proepicardium emerges as a cluster of cells from the septum transversum mesenchyme in close proximity to the heart tube. It gives rise to the outermost layer of the heart, the epicardium, from which cardiac fibroblasts embedded in the myocardium and vascular smooth muscle cells of the coronary vessels are derived (Brade et al., 2013; Xin et al., 2013).

The heart and foregut form in close spatiotemporal proximity; the development of the respective anlagen is interrelated from the earliest stages of cardiogenesis (see poster, ‘Heart development’). Chick embryos have been widely used as a model organism to study this interaction, because heart and foregut development in the chick is similar to that in mammals, including humans (Hosseini et al., 2017; Kirby, 2007). The bilateral heart-forming regions form in close association with the foregut endoderm (Buckingham et al., 2005; Kirby, 2007). The induction and further differentiation of cardiac mesoderm within the heart-forming regions, as well as the establishment of an endocardial layer, require factors derived from the anterior foregut endoderm (AFE) (Brand, 2003; Marvin et al., 2001; Sugi and Lough, 1995; Sugi and Markwald, 2003). Later in development, endodermal folding leads to closure of the foregut; consequently, the heart-forming regions are brought in close proximity to each other and fuse into the heart tube (Kirby, 2007). Although heart development requires AFE-derived signals, factors emitted by the cardiac mesoderm pattern the foregut by inducing lung and liver formation within the AFE and posterior foregut endoderm (PFE), respectively (see poster, ‘Heart development’) (Jung et al., 1999; Serls et al., 2005).

Novel technologies such as single-cell RNA sequencing (scRNAseq) have considerably improved our understanding of heart development over the past decade. scRNAseq has enabled the analysis of single-cell transcriptomes at high resolution and throughput. This has helped to identify novel or rare cardiac cell populations, to elucidate developmental mechanisms and cell lineage trajectories during cardiac specification and differentiation, and to generate single-cell atlases to map cardiac cell types at different developmental stages in animal models (DeLaughter et al., 2016; Gonzalez et al., 2022; Li et al., 2016; Paik et al., 2020; Tyser et al., 2021), which can now be used to better specify hPSC-based *in vitro* models.

## Cardiac differentiation strategies

During the past years, researchers have made considerable progress in the efficient differentiation of hPSCs into cardiomyocytes *in vitro*. The general idea behind such directed differentiation strategies is to mimic the key steps of early cardiogenesis known from *in vivo* developmental studies by applying growth factors or small molecules (Kempf and Zweigerdt, 2018) (see poster, ‘Cardiac differentiation strategies’). Following the cardiac developmental process, hPSCs are directed through primitive streak/mesoderm induction over cardiac mesoderm specification to cardiomyocyte formation. Particularly, the biphasic control of the canonical WNT signaling pathway has been shown to play a major role. WNT signaling is activated in mesoderm induction during gastrulation and inhibited during cardiac mesoderm specification (Arnold et al., 2000; Ueno et al., 2007).

During gastrulation, BMP4 signaling activates the WNT pathway, which in turn induces NODAL signaling; together, this triggers the expression of mesendodermal markers such as brachyury (also known as TBXT), EOMES and MIXL1 in the prospective primitive streak (Arnold et al., 2000; Ben-Haim et al., 2006; Martyn et al., 2018; Kempf et al., 2016). Consequently, to mimic primitive streak and mesoderm induction *in vitro*, different protocols apply BMP4, activin A and WNT3A (Elliott et al., 2011; Kattman et al., 2011; Laflamme et al., 2007; Yang et al., 2008). Alternatively, the formation of primitive streak-like cells can be efficiently induced using the small molecule CHIR99021, which acts as a WNT pathway activator (Gonzalez et al., 2011; Lian et al., 2013).

Following primitive streak induction in the embryo, brachyury and EOMES initiate the expression of the key cardiac regulator MESP1 (David et al., 2011a,b) in the pre-cardiac mesoderm, which in turn drives cardiac specification through DKK1-mediated WNT pathway inhibition (David et al., 2008) and regulates the downstream cardiac gene network, including the expression of genes encoding NKX2.5 (also known as NKX2-5), GATA4, TBX5 and ISL1 (Burridge et al., 2012). Additionally, cardiac specification requires signals from the adjacent AFE, including BMPs, FGFs and WNT inhibitory factors such as crescent and DKK1 (Marvin et al., 2001; Sugi and Lough, 1995). Accordingly, DKK1-mediated WNT pathway inhibition has been successfully used to specify cardiac mesoderm following mesoderm induction *in vitro* (Yang et al., 2008). Small molecules that inhibit the WNT pathway, such as IWP, IWR1 and Wnt-C59, efficiently replace DKK1, resulting in improved differentiation outcomes in both two-dimensional (2D) and three-dimensional (3D) culture (Minami et al., 2012; Willems et al., 2011; Burridge et al., 2014; Halloin et al., 2019; Kempf et al., 2014, 2015, 2016; Lian et al., 2013).

In its simplest form, biphasic modulation of canonical WNT signaling results in efficient differentiation of predominantly ventricular-like cardiomyocytes (Burridge et al., 2014; Halloin et al., 2019; Kempf et al., 2015). Extended protocols have been developed to specify further cardiac subtypes such as atrial-like (Devalla et al., 2015) or pacemaker-like (Protze et al., 2017) cardiomyocytes. Moreover, different groups have established the differentiation of additional cardiac cell types from hPSCs, including endothelial cells and smooth muscle cells (Olmer et al., 2018; Orlova et al., 2014; Patsch et al., 2015), as well as epicardial cells and epicardium-derived fibroblasts and smooth muscle cells (Bao et al., 2017; Guadix et al., 2017; Witty et al., 2014). The knowledge derived from the generation of these different cell types lays the foundation for the formation of cardiac *in vitro* models.

## *In vitro* models of heart tissue, development and disease

Over the past few years, a wide range of simple cardiomyocyte-only 2D and 3D platforms have been used to model different cardiac diseases (Chatterjee et al., 2021; de la Roche et al., 2019; Giacomelli et al., 2017b; Kim et al., 2013). In parallel, researchers have established more complex multicellular 3D models of the heart, which more closely mimic heart development or the cellular composition and physiology of the adult heart, and which have therefore been successfully applied for disease modeling and drug screening. In the following paragraphs, we provide an overview of such models, which we categorize into cardiac organoids, cardiac microtissues and engineered cardiac tissues, respectively (Box 1, Table 1, poster, ‘Cardiac *in vitro* models: nomenclature’). However, despite the classification we applied here for systematic reasons, we will refer to the nomenclature used in the respective original publications throughout this article (highlighted by quotation marks) to respect the authors' original work.

**Box 1. Nomenclature of cardiac *in vitro* models**

**Cardiac organoids.** In general, organoids are 3D structures that resemble the morphology, cellular composition, functionality and development of native organs. They can be derived from pluripotent stem cells (PSCs) or adult stem cells, which are exposed to tissue-specific differentiation factors and often encapsulated in a hydrogel such as Matrigel. Importantly, during the differentiation process, the cells self-organize into embryo-like tissue patterns according to the same organizing principles known from the organ itself (Clevers, 2016; Fatehullah et al., 2016; Lancaster and Huch, 2019). Accordingly, cardiac organoids are PSC-derived self-organizing spheroids that resemble the key features of heart development and tissue structure. Therefore, cardiac organoids represent a model for the embryonic or fetal heart and the underlying developmental processes, rather than mimic adult-like heart tissue.

**Cardiac microtissues.** Cardiac microtissues are generated by the co-aggregation of multiple cardiac cell types in 3D culture. Here, pre-differentiated or primary cardiac cell types such as cardiomyocytes, endothelial cells and fibroblasts are combined into a spheroid. Cardiac microtissues show limited self-organization and pattern formation, and do not recapitulate cardiac development, but serve as a potent model for adult-like heart tissue.

**Engineered cardiac tissues.** Engineered cardiac tissues are generated by combining pre-differentiated or primary cardiac cell types such as cardiomyocytes, endothelial cells and fibroblasts with a hydrogel, serving as a scaffold. Typically, such hydrogels are based on collagen, fibrin or Matrigel. Engineered cardiac tissues display limited self-organization and patterning, and do not aim at recapitulating cardiac development, but at resembling adult-like heart tissue structure and properties. Maturation of engineered cardiac tissues has been achieved by different methods, such as mechanical stretch or electrical stimulation. Unlike cardiac microtissues and organoids, engineered cardiac tissues are typically forced into their 3D shape, for example by using molding technologies combined with complex bioreactors or simplified multi-well-based mechanical setups.

**Table 1.**
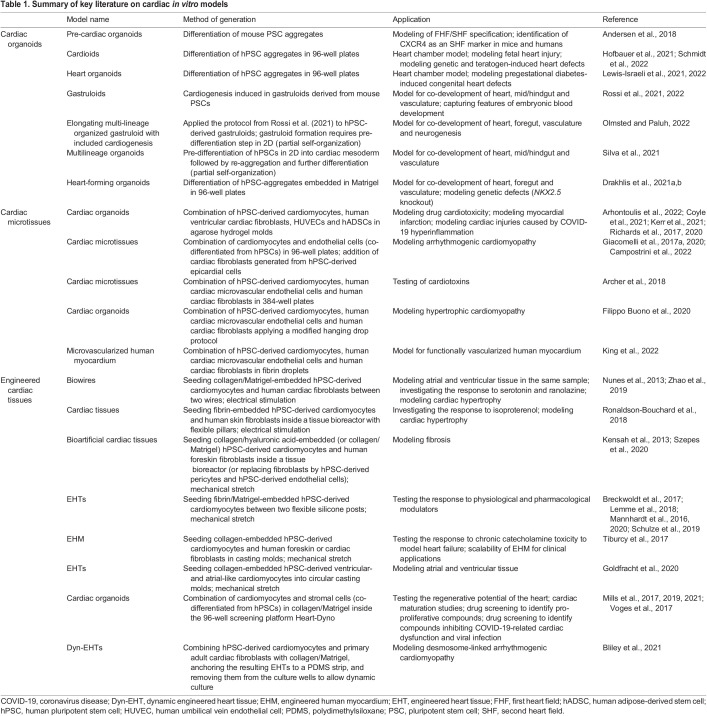
Summary of key literature on cardiac *in vitro* models

## Cardiac organoids

Although tissue-specific primary cells or hPSCs have been successfully used for over a decade to generate organoid models for a broad range of tissues including the intestine (Sato et al., 2009), brain (Lancaster et al., 2013) and kidney (Takasato et al., 2015), organoids that recapitulate proper cardiogenesis have only been derived very recently. Cardiac organoids represent a complex, *in vivo*-like developmental model with multiple cell lineages forming via self-organization and as a consequence of controlled PSC differentiation approaches. Therefore, cardiac organoids are potent tools for modeling and investigating aspects of heart development and disease; moreover, cardiac organoids produced in a 96-well format provide a suitable screening platform for drug development and for monitoring the teratogenic potential of different substances. An important limitation of cardiac organoids is that they mainly resemble embryonic or fetal heart tissue; thus, their capacity for modeling mature heart tissue and respective diseases typically occurring during adulthood is limited.

Additionally, current cardiac organoid models fail to recapitulate processes such as cardiac looping, proper chamber formation including septation and valvulogenesis, trabeculation and outflow tract patterning. Consequently, a large part of congenital malformations resulting from defects in these processes cannot be properly modelled by the available organoids. Moreover, the generation of organoids often requires complex protocols with little control over the cell composition and morphology, which introduces some unpredictability to these systems (Lancaster and Huch, 2019). The cardiac organoid models developed to date can be divided into two groups, organoids mimicking the heart only and more complex multi-tissue organoids (see poster, ‘Cardiac organoids’).

Andersen et al. used mouse PSCs to form ‘precardiac organoids’, in which FHF and SHF cells developed in two distinct areas, showing high similarities to their *in vivo* counterparts (Andersen et al., 2018). Although simple in nature, these organoids provided novel insights into heart field specification. Moreover, they were used to identify the chemokine receptor CXCR4 as a surface marker of SHF cells in mice and humans (Andersen et al., 2018), which enables the isolation of these cells irrespective of reporter transgenes.

Hofbauer et al. used hPSCs in a 96-well format to derive chamber-like ‘cardioids’. These structures contain separate myocardial and endocardial lineages, resembling the heart tube stage (Hofbauer et al., 2021). Co-culture of cardioids with epicardial aggregates to simulate the formation of the proepicardium induced the interaction of cardioids with epicardial cells, which resembles aspects of early heart chamber development. The authors used the cardioids to model fetal heart injury, revealing cell-type-dependent accumulation of extracellular matrix proteins upon cryoinjury. Limitations of this organoid model include the presence of only FHF cells but no SHF progenitors; moreover, the chambers seem to form by fusion of smaller chambers, which develop shortly after mesoderm induction, representing differences to *in vivo* development. An impressive follow-up preprint describes the generation of cardioids for all major embryonic heart compartments, which functionally connected to form multi-chamber cardioids with a shared lumen. The authors successfully used these cardioids to model genetic and teratogen-induced heart defects *in vitro* (Schmidt et al., 2022 preprint).

Lewis-Israeli et al. generated fully self-organized, hPSC-derived ‘heart organoids’ in a 96-well format containing myocardial tissue interspersed with fibroblasts, interconnected small chambers lined with endocardial cells, vasculature and epicardial clusters (Lewis-Israeli et al., 2021, 2022). Moreover, these heart organoids recapitulated aspects of FHF and SHF formation, as well as atrioventricular specification. The authors used their organoids to model pregestational diabetes-induced congenital heart defects, showing structural, metabolic and functional effects on organoids cultured under diabetic conditions.

Recently, several groups have described multi-tissue organoids that mimic the co-development of cardiac and gut lineages. Although the presence of non-cardiac tissues may seem to complicate cardiac-specific studies and applications, such multi-tissue organoids provide the unique opportunity to model mesoderm–endoderm interactions known from native development and properly analyze the effects of genetic or pharmacological perturbation on several tissues simultaneously.

Rossi et al. have described a method for inducing cardiogenesis in gastruloids (Baillie-Johnson et al., 2015) derived from mouse PSCs within 7 days (Rossi et al., 2021). These organoids comprise cardiac progenitors self-organizing into a cardiac crescent- and heart tube-like domain, which is separated from a primitive gut-like tube by an endocardial layer. Moreover, they show an anterior–posterior polarity reminiscent of the embryonic axis, formation of FHF and SHF progenitors and a vascular network. Although these organoids closely recapitulate patterns of early cardiogenesis, one major limitation of this model is the formation of mid/hindgut tissue, as identified by expression of the marker CDX2, instead of foregut endoderm as in native heart development. A follow-up study captured features of embryonic blood development in these gastruloids, increasing the complexity and value of this multi-tissue model (Rossi et al., 2022). Olmsted and Paluh applied the protocol from Rossi et al. (2021) to their human gastruloids, achieving a combined model for cardiogenesis, foregut development and neurogenesis (Olmsted and Paluh, 2022). However, these organoids were not fully self-organized because they required a pre-differentiation step in 2D prior to re-aggregation in 3D and further differentiation.

Similarly, Silva et al. published a heart–mid/hindgut organoid model derived from hPSCs, which were pre-differentiated in 2D into cardiac mesoderm followed by re-aggregation, further differentiation and self-organization for 30–100 days (Silva et al., 2021). The resulting ‘multilineage organoids’ contained a cardiac core of mainly atrial-/nodal-like cells encircled by gut-like tissue and epicardial cells, and persisted in culture for more than a year. However, the cardiac core did not resemble its *in vivo* counterpart, and the epicardial layer surrounded the entire organoid and not just the cardiac tissue, as it does *in vivo*. Using these organoids, the authors showed that co-development with endoderm can increase the maturation of cardiomyocytes *in vitro*, consistent with *in vivo* observations showing that signals from the endoderm are required for proper heart formation (Sugi and Lough, 1995).

Our own group has established a 14-day protocol for the generation of ‘heart-forming organoids’ (HFOs) from hPSCs in a 96-well format (Drakhlis et al., 2021a,b). Although HFOs are fully self-organized without requiring a pre-differentiation step, the strategy depends on Matrigel, an undefined extracellular matrix preparation. HFOs are highly organized structures containing an SHF-derived myocardial layer separated by an endocardial-like layer from an inner core comprising AFE tissue. The myocardial layer is surrounded by an outer layer composed of septum-transversum-like cells, mesenchymal cells, cardiomyocytes and liver tissue (representing PFE). Moreover, HFOs are pervaded by a vascular network. The morphology of HFOs resembles the early heart–foregut region prior to heart tube formation in the developing embryo. Limitations of the model include the lack of FHF cells and the presence of two spatially independent foregut endoderm compartments instead of a single foregut tube. Using HFOs, we showed that the deletion of the cardiac transcription factor NKX2.5 caused defects in the myocardial layer reminiscent of malformations known from transgenic mice (Lyons et al., 1995; Pashmforoush et al., 2004), such as a decreased adhesion between cardiomyocytes and cardiac hypertrophy.

Researchers have developed a diverse array of cardiac organoids with different degrees of complexity. Although the capability of these organoids to resemble the human heart is limited at present, they do provide a valuable modeling platform for cardiac development and disease.

## Cardiac microtissues

The main aim for using cardiac microtissues is to mimic the cellular composition of human adult-like heart tissue and to achieve an increased cardiomyocyte maturation by combining pre-differentiated or primary cardiac cell types (see poster, ‘Cardiac microtissues’). Although microtissues have limited ability to recapitulate heart development and morphogenesis due to restricted self-organization and patterning, these models show a number of advantages. These include the relative simplicity of generation and handling of the system, and the relatively high control over cell composition, size and shape of the microtissues, resulting in a higher reproducibility, which makes them suitable for high-throughput-screening approaches.

Richards et al. combined hPSC-derived cardiomyocytes with primary human ventricular cardiac fibroblasts, human umbilical vein endothelial cells (HUVECs) and human adipose-derived stem cells (hADSCs) in agarose hydrogel molds to generate ‘cardiac organoids’ that resembled the structure and functionality of the vascular network in the developing myocardium (Richards et al., 2017). These microtissues were used to evaluate the cardiotoxicity of drugs and to model myocardial infarction by application of an oxygen diffusion gradient and noradrenaline stimulation (Richards et al., 2020). Follow-up studies showed that this model was transcriptomically similar to adult myocardium (Kerr et al., 2021) and that its vascularization could be increased by the upregulation of the hypoxia-inducible factor HIF-α (Coyle et al., 2021). Furthermore, these microtissues were used to model cardiac injuries caused by COVID-19 hyperinflammation through stimulation with IL-1β to mimic a cytokine storm (Arhontoulis et al., 2022).

Giacomelli et al. co-differentiated cardiomyocytes and endothelial cells from hPSCs in 2D monolayer culture and used these cells to form 3D ‘cardiac microtissues’ in a 96-well format (Giacomelli et al., 2017a). In this culture system, the cardiomyocytes showed a higher expression of maturation-associated genes compared to microtissues composed of cardiomyocytes alone, highlighting the beneficial effect of endothelial cells on the cardiomyocytes' maturation status. The addition of cardiac fibroblasts to the microtissues further enhanced cardiomyocyte maturation, and, when using patient-derived fibroblasts, the microtissues modeled aspects of arrhythmogenic cardiomyopathy (Giacomelli et al., 2020). Notably, the cardiac fibroblasts were generated from hPSC-derived epicardial cells following the *in vivo* developmental path. In contrast to these fibroblasts, adult skin fibroblasts did not increase cardiomyocyte maturation, highlighting how important the developmental origin of the cells is for the functionality of the model.

Campostrini et al. used the same type of microtissues (Giacomelli et al., 2020) but applied patient-derived hiPSC-derived cardiomyocytes carrying mutations in the adult isoform of the cardiac sodium channel SCN5A, which undergoes a fetal-to-adult isoform switch around birth (Campostrini et al., 2022). So far, mutations in the adult isoform have not been captured by conventional hPSC-derived cardiomyocyte cultures due to their fetal phenotype. By contrast, the advanced maturation phenotype in the hiPSC-derived microtissues promoted the SCN5A isoform switch, suggesting the applicability of this model to examine post-natal cardiac arrhythmias (Campostrini et al., 2022).

Three groups have generated cardiac microtissues by combining hPSC-derived cardiomyocytes with primary human cardiac microvascular endothelial cells and human cardiac fibroblasts. Archer et al. used their microtissues to assess their suitability for testing of cardiotoxins (Archer et al., 2018), while Filippo Buono et al. used hiPSC-derived cardiomyocytes from healthy versus diseased patients to model hypertrophic cardiomyopathy (Filippo Buono et al., 2020). King et al. encapsulated the respective cell types in fibrin droplets, thereby generating a functionally vascularized *in vitro* model of the human myocardium (King et al., 2022). We consider this model an intermediate between cardiac microtissues and engineered cardiac tissue, which we describe in more detail below.

## Engineered cardiac tissues

Similar to microtissues, engineered cardiac tissues aim at mimicking the cellular composition and functionality of the adult human heart by combining pre-differentiated or primary cardiac cell types (see poster, ‘Engineered cardiac tissues’). Most of these models consist of hydrogel-embedded cells forming strip- or ring-shaped structures showing hallmarks of cardiac maturation induced by mechanical or electrical stimulation. Engineered cardiac tissues are suitable models for contractile force and kinetic measurements. Limitations of this model include the laborious protocols for generation of the tissues, typically requiring special equipment for tissue molding and often complex bioreactor setups, which limit its suitability for screening approaches despite some progress towards the latter (Mannhardt et al., 2020; Mills et al., 2019).

Several laboratories have pioneered this field using neonatal rat or embryonic chick cardiomyocytes (Carrier et al., 1999; Eschenhagen et al., 1997; Hansen et al., 2010; Radisic et al., 2004). Nowadays, several approaches using hPSCs are available, as described below.

The Radisic laboratory developed ‘biowires’ by seeding hydrogel-embedded hPSC-derived cardiomyocytes and human adult cardiac fibroblasts between two wires to electrically stimulate the resulting tube-like tissues (Nunes et al., 2013). The laboratory then generated biowires with distinct atrial- and ventricular-like regions that could be studied in the same sample (Zhao et al., 2019). Using this advanced platform, the authors measured spatially confined responses to serotonin and the rapid voltage-gated Na^+^ channel blocker ranolazine, which are known to preferentially affect atrial cardiomyocytes. Accordingly, both substances showed effects on the atrial but not the ventricular end of the biowires. Moreover, using patient-derived cells, biowires modeled some aspects of left ventricular hypertrophy (Zhao et al., 2019).

Ronaldson-Bouchard et al. combined hPSC-derived cardiomyocytes with human skin fibroblasts and fibrin within a tissue bioreactor with flexible pillars. After the cardiac tissues formed around these pillars, the tissues were treated with gradually increasing electrical stimulation to achieve adult-like maturation. The resulting engineered tissues showed physiological responses to the β-blocker isoproterenol and recapitulated pathological hypertrophy (Ronaldson-Bouchard et al., 2018).

The Gruh laboratory used hPSC-derived cardiomyocytes and human foreskin fibroblasts to generate bioartificial cardiac tissues subjected to mechanical stress in a custom-made bioreactor (Kensah et al., 2013). These models achieved contractile forces comparable to those of native myocardium. Replacing fibroblasts with hPSC-derived pericytes led to a fibrosis-like phenotype in the bioartificial cardiac tissues, which was even more pronounced when hPSC-derived endothelial cells were added to form the tissues (Szepes et al., 2020).

Seeding hPSC-derived cardiomyocytes in fibrin/Matrigel between two flexible silicone posts resulted in strip-shaped ‘engineered heart tissues’ (EHTs) that generated force under auxotonic stretch conditions (Breckwoldt et al., 2017; Mannhardt et al., 2016, 2020). Contractility analysis revealed that the EHTs showed *in vivo*-like responses to a range of physiological and pharmacological modulators, highlighting their applicability for preclinical drug testing. In follow-up studies, Lemme and colleagues generated chamber-specific atrial EHTs (Lemme et al., 2018), and Schulze and colleagues used them to dissect pacemaker function (Schulze et al., 2019).

Tiburcy et al. generated ring-shaped ‘engineered human myocardium’ from hPSC-derived cardiomyocytes and human foreskin or cardiac fibroblasts that displayed structural and functional properties of postnatal myocardium. These showed an *in vivo*-like response to chronic catecholamine toxicity, resembling classical signs of heart failure. Furthermore, the authors demonstrated the scalability of these models (Tiburcy et al., 2017), opening new perspectives for clinical applications. Similar ring-shaped EHTs were also generated by Goldfracht et al. using ventricular- and atrial-like hPSC-derived cardiomyocytes embedded in collagen to create chamber-specific tissues (Goldfracht et al., 2020).

The Hudson group developed ‘cardiac organoids’ by pre-differentiating hPSCs in 2D, resulting in a mixture of cardiomyocytes and stromal cells, which were subsequently embedded in collagen/Matrigel within a 96-well screening platform termed Heart-Dyno. Following partial self-organization, the resulting structures recapitulated several aspects of heart tissue, including stromal cells, an endothelial network and an epicardial layer. Applying this model system, the group revealed an endogenous regenerative potential following cryoinjury (Voges et al., 2017), showed that a switch to fatty acid metabolism was crucial for cardiac maturation (Mills et al., 2017) and identified compounds promoting cardiomyocyte proliferation by drug screening (Mills et al., 2019). Finally, the authors identified compounds that could inhibit COVID-19-related inflammation-induced cardiac dysfunction and viral infection (Mills et al., 2021).

Bliley et al. developed strip-shaped ‘dynamic EHTs’ (dyn-EHTs) by mixing hPSC-derived cardiomyocytes and primary adult cardiac fibroblasts with collagen/Matrigel, anchoring the resulting EHTs to polydimethylsiloxane (PDMS) strips, and culturing them in suspension to better recapitulate aspects of tissue preload (stretch during ventricular filling) and afterload (pressure the heart must contract against to eject blood), resulting in improved contractile function. (Bliley et al., 2021). In contrast to conventional engineered cardiac tissue models, the dyn-EHTs enabled modeling aspects of desmosome-linked arrhythmogenic cardiomyopathy with patient-derived hiPSC-derived cardiomyocytes (Bliley et al., 2021).

Together, these examples demonstrate that, despite some limitations, engineered cardiac tissues represent a potent platform for drug testing and disease modeling on adult-like heart tissue *in vitro*.

## Conclusions

Mammalian cardiogenesis is a complex process involving the coordinated interaction of multiple different progenitor cells that eventually give rise to the three-layered, four-chambered heart. Modeling this process has proven challenging; however, this field has achieved considerable progress in the past decade. At present, different methods are available to model heart development, morphology and disease *in vitro*. By adding a more structured classification to the heterogeneous nomenclature of different *in vitro* models, our aim was to provide a current overview of cardiac organoids, cardiac microtissues and engineered cardiac tissues. Each of these modeling systems has advantages and limitations; it is therefore important to understand both the overarching concepts and the individual properties of current *in vitro* cardiac models to choose the most appropriate platform for addressing the specific scientific questions in hand. In this regard, researchers should consider which aspect of cardiac development, physiology or pathology the respective approach can effectively model, as well as the complexity of the protocol and scalability of the method.

Cardiac microtissues and engineered cardiac tissues provide potent tools to recapitulate adult-like heart tissue structure and function, which is of utmost importance for modeling diseases of the adult heart and for drug discovery, but they do not model heart development and often lack the overall structural complexity of native heart tissue, such as endocardial, myocardial and epicardial layer formation. By contrast, cardiac organoids represent a complex *in vivo*-like model, making them a suitable platform for studying developmental processes and a variety of congenital disorders, as well as for teratogenicity assessment of substances affecting early embryonic stages. Notably, cardiac organoids are mostly limited to modeling early embryonic or fetal tissue and still lack the much higher complexity of the native heart, including, for example, the trabeculae, the conduction system, heart valves or a distinct arteriovenous vessel structure and a pulsatile blood flow.

Therefore, we envision that, in the near future, researchers will focus on combining self-organization in cardiac organoids with tissue engineering approaches. This chimeric approach aims at refining and expanding the structure and complexity of cardiac organoid models, including their progressive maturation to resemble later developmental stages of the fetus more closely, before focusing on modeling the adult heart in the upcoming future. Together, this will substantially advance the current perspectives for drug discovery and modeling heart development and disease beyond the fetal stage.

## Poster

Poster
