# Supplementary figures and images for: Heart in a dish – choosing the right in vitro model
Source: Dis Model Mech. 2023 Feb 24;16(5):dmm049961. doi: 10.1242/dmm.049961 (PMC9985945; doi:10.1242/dmm.049961)

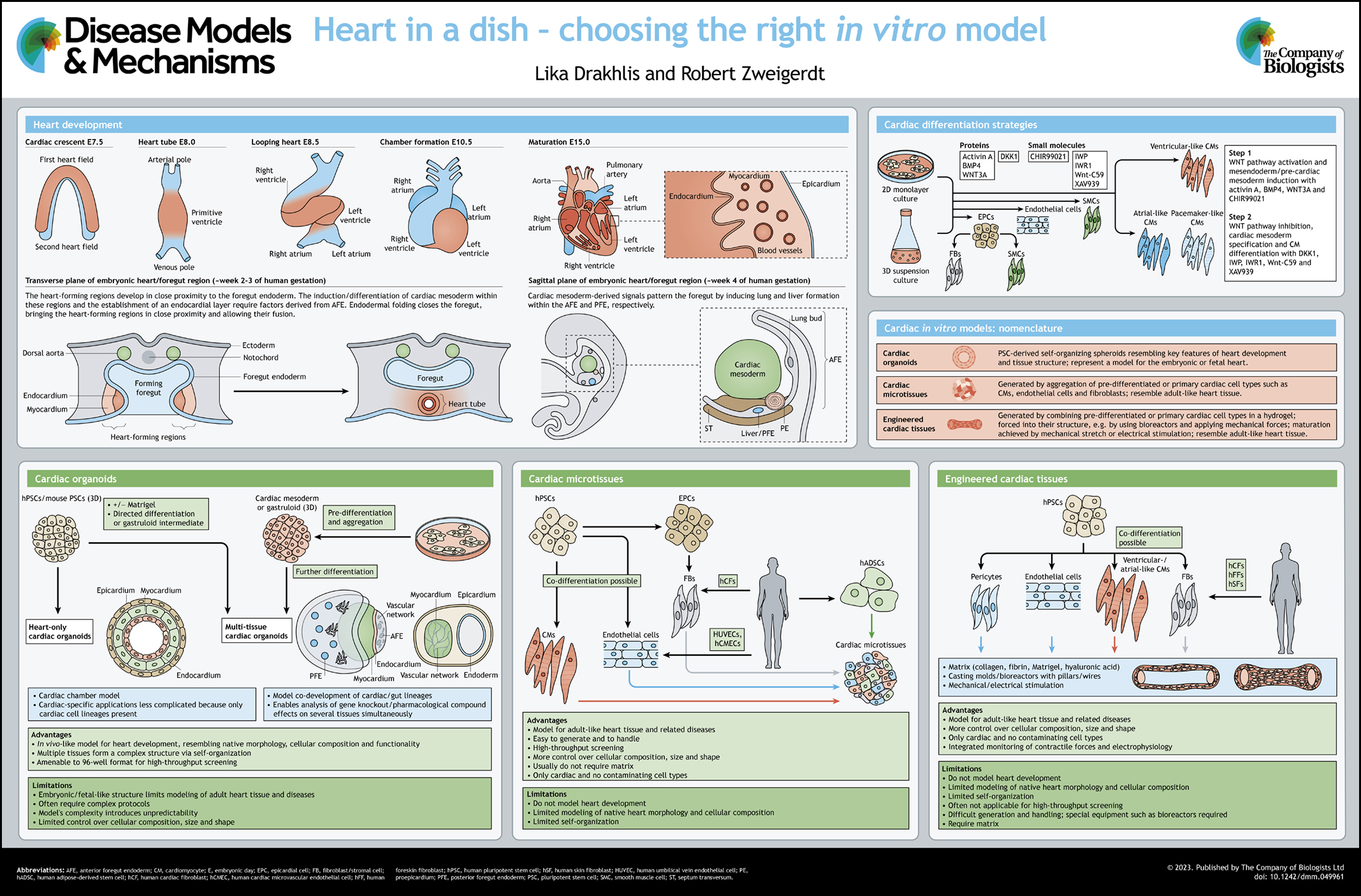

Supplement: Poster [file dmm-16-049961-s1.jpg]
